# Advancing genetic engineering with active learning: theory, implementations and potential opportunities

**DOI:** 10.1093/bib/bbaf286

**Published:** 2025-07-08

**Authors:** Qixiu Du, Haochen Wang, Benben Jiang, Xiaowo Wang

**Affiliations:** Ministry of Education Key Laboratory of Bioinformatics, Center for Synthetic and Systems Biology, Beijing National Research Center for Information Science and Technology, Tsinghua University, No. 1 Qinghuayuan Street, Haidian District, Beijing 100084, China; Department of Automation, Tsinghua University, No. 1 Qinghuayuan Street, Haidian District, Beijing 100084, China; Ministry of Education Key Laboratory of Bioinformatics, Center for Synthetic and Systems Biology, Beijing National Research Center for Information Science and Technology, Tsinghua University, No. 1 Qinghuayuan Street, Haidian District, Beijing 100084, China; Department of Automation, Tsinghua University, No. 1 Qinghuayuan Street, Haidian District, Beijing 100084, China; Department of Automation, Tsinghua University, No. 1 Qinghuayuan Street, Haidian District, Beijing 100084, China; Ministry of Education Key Laboratory of Bioinformatics, Center for Synthetic and Systems Biology, Beijing National Research Center for Information Science and Technology, Tsinghua University, No. 1 Qinghuayuan Street, Haidian District, Beijing 100084, China; Department of Automation, Tsinghua University, No. 1 Qinghuayuan Street, Haidian District, Beijing 100084, China; College of Artificial Intelligence, Tsinghua University, No. 1 Qinghuayuan Street, Haidian District, Beijing 100084, China

**Keywords:** genetic engineering, machine learning, uncertainty, acquisition function, biological fitness landscape

## Abstract

Employing machine learning (ML) models to accelerate experimentation and uncover biological mechanisms has been a rising tendency in genetic engineering. However, effectively collecting data to enhance model accuracy and improve design remains challenging, especially when data quality is poor and validation resources are limited. Active learning (AL) addresses this by iteratively identifying promising candidates, thereby reducing experimental efforts while improving model performance. This review highlights how AL can assist scientists throughout the design-build-test-learn cycle, explore its various practical implementations, and discuss its potential through the integration of cross-domain expertise. In the age of genetic engineering revolutionized by data-driven ML models, AL presents an iterative framework that significantly enhances the functionalities of biomolecules and uncovers their intrinsic mechanisms, all while minimizing expenses and efforts.

## Introduction

The pursuit of designing biological systems with enhanced functionalities (i.e. fitness) [1] has gained increasing popularity in medicine, agriculture, and bio-manufacturing. This highlights the importance of genetic engineering, the domain specializing in designing genetic sequences to alter, repair or enhance functionalities [2]. Recently, advancements in machine learning (ML) have significantly advanced genetic engineering, enabling the precise and efficient engineering of biological elements that target functions not naturally observed through the lengthy process of evolution. ML has been widely employed in various genetic engineering implementations, including prediction of protein structure [3] and epigenetic modification [4], inference of gene networks [5, 6], virtual screening of small-molecule drugs [7, 8], and optimization of programmable ribonucleic acid (RNA) switches [9, 10], CRISPR-Cas systems [11], chimeric antigen receptor (CAR) T cells [12] and adeno-associated virus (AAV) capsids [13, 14], etc.

While ML method provides valuable insights from data that are traditionally hard to analyze, their effectiveness relies on certain mathematical constraints. ML-assisted approaches typically operate under closed-world assumptions, expecting that unexplored candidates exhibit the similar underlying patterns to the training data—a condition known as in-distribution assumption [15, 16]. However, the natural biological landscape is often complex and uneven, which makes it challenging to construct globally reliable predictive models. One possible explanation is that biological mutations in the nature are typically random. Even within the homogeneous environment, different mutations can accumulate independently based on historical contexts, leading to distinct evolutionary paths. This process allows species to evolve along these unique trajectories, resulting in the coexistence of various genotypes in the same environment [17]. This indicates that limited-scale training datasets of naturally evolved genotypes cover only a small fraction of complex genetic information, thus they often do not conform to the global distribution, violating the closed-world assumption of ML-assisted approaches. This intrinsic complexity of biological data, along with the constraints of experimental techniques, have presented three major challenges to the effective application of ML methods:

One of the biggest challenges is the difficulty in systematically exploring the underlying relationships between biological sequence and fitness (i.e. the fitness landscape). On the one hand, candidate sequences grow exponentially in size as the sequence length ($L$) increases, reaching ${4}^L$ for DNA/RNA and ${20}^L$ for protein. On the other hand, the intrinsic regulatory patterns lie in biological sequences are rather complex. For instance, the combinatorial mutational effects of multiple nucleotides/amino acids are typically non-additive (i.e. epistatic effects), combination of mutations that individually improve fitness may not necessarily lead to further fitness improvement [18–22]. Therefore, current available biological data are often biased in local, with only a few validations on specific designs, failing to fully represent the overall vast and complex relationships between sequences and their fitness. These factors make ML models prone to overfitting, leading to false predictions for unexplored candidates (i.e. Out-of-Distribution) [23].

Second, the fitness value typically follows a long-tailed distribution, implying that most genotypes are nonfunctional, while only a very small fraction of elements exhibit the desired activities in target functions. For instance, the combinatorial libraries of regions critical to protein function tend to be enriched in zero- or extremely low-fitness variants, like Protein G Domain B1 domain (of all the combination of 4 amino acids, 92% have fitness below 1% of that of the global maximum) [24] and dihydrofolate reductase folA gene (9 bp, 93% nonfunctional) [25]. Therefore, the training dataset typically consists of scarce functional sequences, thus its fitness distribution is highly imbalanced, constraining the model’s capacity to effectively navigate the fitness landscape.

Third, obtaining a sufficient number of well quantified data to iteratively improve the design is often constrained, as biological experiments are often expensive and time-consuming. For instance, while single-cell sequencing technology has become relatively affordable [26], its cost still limits large-scale studies of cellular expression responses to genetic perturbations [27]. Therefore, the available experimental validations in genetic engineering are often limited, providing insufficient feedback to generate new insights for optimizing the design or improving ML models.

The complexity of biological sequences and the limitations of experimental scale have constrained the application of ML models in genetic engineering. This raises an important question: what qualities should an ML framework possess to effectively meet the practical needs of genetic engineering? Due to the aforementioned inherent complexity of natural biological systems, training an ML model solely on the initial training set and sampling up to the cost-permitted scale within a single iteration is prone to result in considerable inaccuracies. Conversely, distributing limited validation resources across multiple iterations, and selecting relatively a small number of candidates that are most effective for improving model performance in each design-build-test-learn (DBTL) cycle has proven to be more practically feasible [28]. An ML framework called active learning (AL) has been introduced to efficiently drive DBTL cycles.

The AL strategy, initially introduced by Lewis *et al.* [29], is designed to achieve higher accuracy with fewer labelling efforts compared to passive learning [30]. This strategy automatically scores and selects promising candidates for validation, followed by training an improved ML model. It not only takes into account the predicted fitness, but also the uncertainty associated with the current ML model predictions. Therefore, AL enables more informed decision-making and has been widely applied in various domains, including the discovery of novel materials with desired physical properties [31, 32], optimization of organic chemistry reaction conditions [33, 34], virtual screening of drugs against specific targets [35, 36], optimization of fast-charging protocols for batteries [37], optimization of tasks for dissociating frontoparietal brain networks [38], and the design of gaits for legged robots to adapt to injuries [39], etc. In addition, previous studies [40–42] have systematically evaluated the effectiveness of AL under varying levels of data insufficiency, demonstrating its superior performance over conventional ML approaches.

Recent applications of AL in genetic engineering, such as reducing the need for immunological experiments when predicting epitope mutation effects [43], and directing the enzyme evolution to improve product yield [21], have demonstrated its significant potential. As a promising technique, the success of AL depends on our ability to integrate it effectively within the DBTL cycle. In this review, we provide an organized overview of AL in genetic engineering, including a clear illustration of relevant algorithms and their current implementations, to help researchers select the appropriate algorithm for their specific genetic engineering scenario. Moreover, we explore potential advancements for AL in genetic engineering through the integration of cross-domain research, foundation models and automation techniques. We believe this will offer valuable guidance for researchers designing their own AL-assisted DBTL cycles, and could potentially provide the expertise and innovative insights needed to advance the frontiers of synthetic biology.

## Theoretical foundations of active learning

### Overview

**Figure 1:**
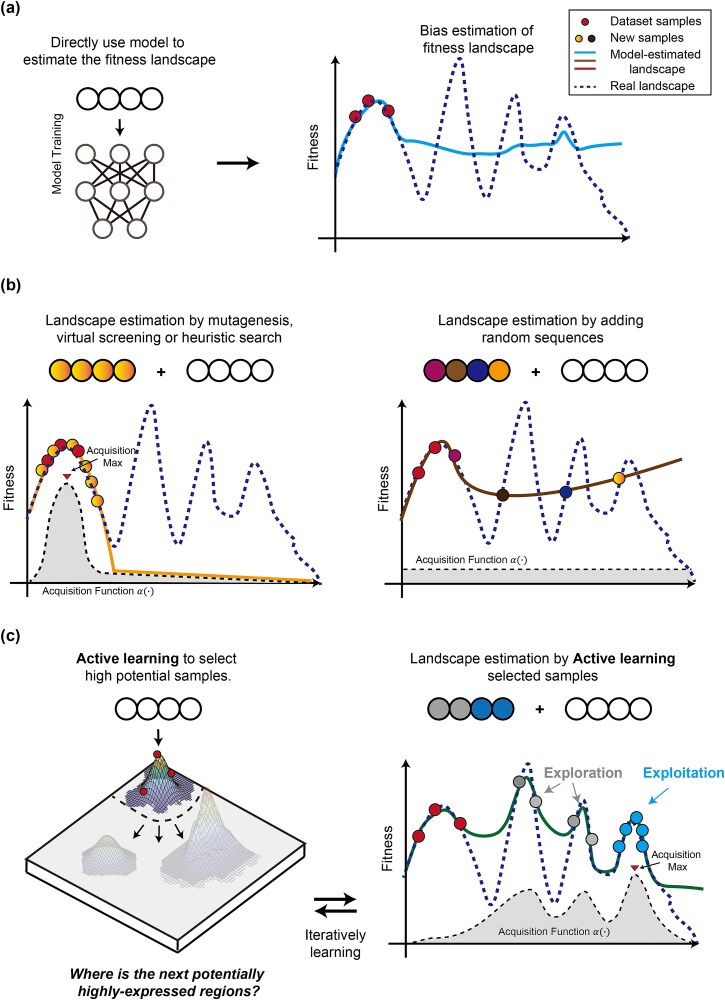
The overview of AL. (a) Sequence landscape estimation based on known dataset. ML models trained on biological samples typically provide low-quality approximations in regions with scarce training data. (b) Sequence landscape estimation based on mutation and random sampling. Traditional sampling approaches might ignore potential promising candidates (e.g. mutagenesis, virtual screening, and heuristic optimization) or suffer from low exploration efficiency (random sampling). (c) Sequence landscape estimation based on AL. AL automatically optimizes models by selecting samples with high predictions or uncertainties, thus enhancing both optimization and exploration efficiency.

Identifying high-fitness genotypes within the fitness landscape is essential for genetic engineering. Traditional ML-assisted DBTL process often fall short in exploration efficiency [44]. The primary reason is that the initially available biological data is typically biased towards certain genotype distributions, causing ML models to provide low-quality approximations for genotypes that deviate from the training data distribution (Fig. 1a). These genotypes, sparsely represented in the training data and appearing as outliers, are challenging to explore due to the limited number of financially feasible trial-and-error attempts (*N*). Currently, there are two distinct design strategies. One strategy is to ignore these outliers and focus resources on exploiting centrally distributed genotypes, which are generally predicted with higher accuracy. This strategy leverages methods such as mutagenesis, virtual screening, and heuristic optimization to explore new designs (Fig. 1b, left). However, this approach can be limiting for more ambitious researchers, as these outlier genotypes may be the tips of the icebergs for potential fitness peaks or novel regulatory patterns that are overlooked. Another strategy is to allocate all resources to exploration (Fig. 1b, right). For instance, Vaishnav *et al.* [45] created a sequence library of size ${10}^8$ by randomly sampling the 80 bp upstream region of yeast promoters. However, this approach is typically inefficient, leading to a large fraction of low-fitness designs, which increases unnecessary labor and costs.

Compared to traditional ML-assisted DBTL cycles, AL-assisted DBTL cycles [46] effectively balance the trade-off between exploration and exploitation (Fig. 1c), by iteratively quantifying the uncertainty of unexplored genotypes and improving model performance with minimal experimentation. Through uncertainty quantifying ($\zeta$) and sampling ($\alpha$) approaches, in each given DBTL cycle $t$, the AL-assisted process can be formulated as follows.


(1) \begin{align*} \left({\boldsymbol{X}}_{\boldsymbol{t}},{\boldsymbol{Y}}_{\boldsymbol{t}}\right)=\left({\boldsymbol{X}}_{\boldsymbol{t}-\mathbf{1}}\cup{\boldsymbol{X}}_{\ast}^{\left(\boldsymbol{t}-\mathbf{1}\right)},{\boldsymbol{Y}}_{\boldsymbol{t}-\mathbf{1}}\cup{\boldsymbol{Y}}_{\ast}^{\left(\boldsymbol{t}-\mathbf{1}\right)}\right) \end{align*}

(2) \begin{align*} \left({\boldsymbol{\mu}}_{\ast}^{\left(\boldsymbol{t}\right)},{\boldsymbol{\sigma}}_{\ast}^{\left(\boldsymbol{t}\right)}\right)=\zeta \left({\boldsymbol{X}}_{\mathbf{cand}},{f}_t|{\boldsymbol{X}}_{\boldsymbol{t}},{\boldsymbol{Y}}_{\boldsymbol{t}}\right) \end{align*}

(3) \begin{align*} {\boldsymbol{X}}_{\ast}^{\left(\boldsymbol{t}\right)}=\arg \underset{{\boldsymbol{x}}_{\ast }}{\max}\alpha \left({\boldsymbol{\mu}}_{\ast}^{\left(\boldsymbol{t}\right)},{\boldsymbol{\sigma}}_{\ast}^{\left(\boldsymbol{t}\right)}\right),{\boldsymbol{Y}}_{\ast}^{\left(\boldsymbol{t}\right)}=\mathrm{BuildTest}\left({\boldsymbol{X}}_{\ast}^{\left(\boldsymbol{t}\right)}\right) \end{align*}

Here ${f}_t\mid{\boldsymbol{X}}_{\boldsymbol{t}},{\boldsymbol{Y}}_{\boldsymbol{t}}$ symbols ML model $f$ trained on genotype-fitness pairs $\left({\boldsymbol{X}}_{\boldsymbol{t}},{\boldsymbol{Y}}_{\boldsymbol{t}}\right)$, ${\boldsymbol{X}}_{\mathbf{cand}}$ symbols the remaining genotypes for exploration, and $\mathrm{BuildTest}$ symbols the building and testing steps of each DBTL cycle. Equation (2) represents uncertainty quantification (UQ), where $\left({\boldsymbol{\mu}}_{\ast}^{\left(\boldsymbol{t}\right)},{\boldsymbol{\sigma}}_{\ast}^{\left(\boldsymbol{t}\right)}\right)$ denote the expectations ($\boldsymbol{\mu}$) and uncertainties ($\boldsymbol{\sigma}$) for all predictions of ${\boldsymbol{X}}_{\mathbf{cand}}$ with strategy $\zeta$. Equation (3) represents the acquisition process, where an acquisition function $\alpha \left(\mu, \sigma \right):{\boldsymbol{X}}_{\mathbf{cand}}\mapsto \mathbb{R}$ is employed to assign a utility value to ${\boldsymbol{X}}_{\mathbf{cand}}$, enabling the selection of potential genotypes ${\boldsymbol{X}}_{\ast}^{\left(\boldsymbol{t}\right)}$ to query next. Note that we use $\arg\ \max$ for clarity, assuming $\alpha$ assigns higher values to preferred samples; if defined oppositely, $\arg\ \min$ applies. In addition, AL can also proceed based on diversity sampling, in which case Equations (2) is not required, and $\alpha$ maps diversity of samples to real values.

By carefully selecting efficient UQ and sampling strategy, AL-assisted DBTL cycles can quickly converge on the true landscape with just a few iterations of small-scale sampling, filtering out a significant portion of unnecessary, costly, and time-consuming validations, and ultimately accelerating the optimization on potential fitness peaks. Although significant reviews of AL-related algorithms have been proposed previously [47], the challenges for biologists remains: these reviews tend to focus on comprehensive summaries of the algorithms, which introduce numerous diverse symbols that complicate interpretation for the research community, and neglect the implementation trends and significance of AL in practical genetic engineering. In the following section, we will provide an organized and unified descriptions of these algorithms, and introduce current significant algorithms in practical engineering.

### Uncertainty quantification

**Figure 2:**
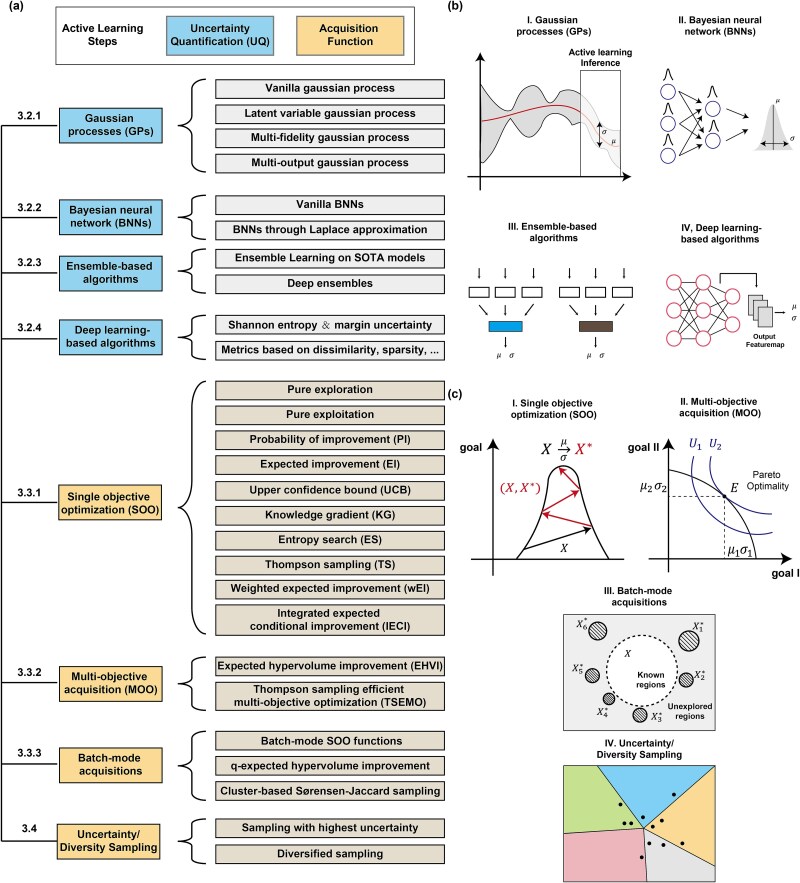
Knowledge graph of AL. (a) Classification of uncertainty quantification algorithms and acquisition functions. (b) Diagrams of current uncertainty quantification approaches. (c) Diagrams of current acquisition functions.

UQ is the process of assessing the predictions and associated confidence levels of ML models, which is essential for avoiding potentially flawed designs. Such flawed designs often arise from irresponsible extrapolations, where the model predicts extraordinarily high fitness that strongly deviate from the general regulatory patterns within biological data. To address this challenge, researchers have developed a series of ML models (e.g. Gaussian processes and Bayesian neural networks) and algorithms (e.g. ensemble-based and deep learning-based) to quantify the uncertainty levels ($\sigma$) when making specific predictions ($\mu$). In the following sections, we discuss these approaches, focusing on their mathematical foundations and current practical applications (Fig. 2).

#### Gaussian process

The Gaussian process (GP) is an ML model that represents the distribution of fitness as an underlying Gaussian distribution. Due to its interpretability and simplicity, GP has become one of the most widely used UQ approaches. The modeling of fitness using GP can be described as Equation (4):


(4) \begin{align*} &f\left(\boldsymbol{x}\right)\sim N\left(\boldsymbol{\mu} \left(\boldsymbol{x}\right),\boldsymbol{K}\left(\boldsymbol{x},\boldsymbol{x}\right)\right)\nonumber\\&\boldsymbol{y}=f\left(\boldsymbol{x}\right)+\boldsymbol{\varepsilon} \sim N\left(\boldsymbol{\mu} \left(\boldsymbol{x}\right),\boldsymbol{K}\left(\boldsymbol{x},\boldsymbol{x}\right)+{\sigma}^2\boldsymbol{I}\right) \end{align*}

Here $f$ represents mappings of genotype $\boldsymbol{x}$ to fitness $\boldsymbol{y}$ estimated by GP, which follows a specific multidimensional Gaussian distribution parametrized by a mean vector $\boldsymbol{\mu} \left(\boldsymbol{x}\right)$ and a covariance matrix $\boldsymbol{K}\left(\boldsymbol{x},\boldsymbol{x}\right)$, while $\boldsymbol{\varepsilon} \sim N\left(0,{\sigma}^2\right)$ symbols the noise term uniformly distributed on all dimensions.

The training process of GP is similar to other ML models, simply fitting $\boldsymbol{\mu} \left(\boldsymbol{x}\right)$ and $\boldsymbol{K}\left(\boldsymbol{x},\boldsymbol{x}\right)$ to the available genotype-fitness pairs $\left(\boldsymbol{X},\boldsymbol{Y}\right)$. When predicting an unexplored genotype ${\boldsymbol{x}}_{\ast}$, the model-estimated distribution $f\left({\boldsymbol{x}}_{\ast}\right)\mid \boldsymbol{X},\boldsymbol{Y}$ follows a multidimensional Gaussian distribution parametrized by predicted mean ${\boldsymbol{\mu}}_{\ast}$ and uncertainty levels ${\boldsymbol{\sigma}}_{\ast}$, which can be inferred analytically [48] (Equation 5).


(5) \begin{align*}& f\left({\boldsymbol{x}}_{\ast}\right)\mid \boldsymbol{X},\boldsymbol{Y}\sim N\left({\boldsymbol{\mu}}_{\ast },{\boldsymbol{\sigma}}_{\ast}^{\mathbf{2}}\right)\nonumber\\ &{\boldsymbol{\mu}}_{\ast }=\boldsymbol{K}\left({\boldsymbol{x}}_{\ast },\boldsymbol{X}\right){\left[\boldsymbol{K}\left(\boldsymbol{X},\boldsymbol{X}\right)+{\sigma}^2\boldsymbol{I}\right]}^{-1}\left(\boldsymbol{Y}-\boldsymbol{\mu} \left(\boldsymbol{X}\right)\right)+\boldsymbol{\mu} \left({\boldsymbol{x}}_{\ast}\right) \nonumber\\& {\boldsymbol{\sigma}}_{\ast}^{\mathbf{2}}=\boldsymbol{K}\left({\boldsymbol{x}}_{\ast },{\boldsymbol{x}}_{\ast}\right)-\boldsymbol{K}\left({\boldsymbol{x}}_{\ast },\boldsymbol{X}\right){\left[\boldsymbol{K}\left(\boldsymbol{X},\boldsymbol{X}\right)+{\sigma}^2\boldsymbol{I}\right]}^{-1}\boldsymbol{K}\left(\boldsymbol{X},{\boldsymbol{x}}_{\ast}\right) \end{align*}

The sound mathematical foundation of GP has endowed it with multiple advantages. For instance, the kernel functions $\boldsymbol{K}\left(\boldsymbol{X},{\boldsymbol{x}}_{\ast}\right)$ inherently quantify the contribution of training data $\boldsymbol{x}\in \boldsymbol{X}$ to prediction $f\left({\boldsymbol{x}}_{\ast}\right)$ [49], thus enhancing interpretability. The commonly used kernels include the linear kernel, polynomial kernel, Gaussian kernel, Laplacian kernel, etc. Specifically, Matérn kernels represent a class of stationary kernels that are shift-invariant, characterized by a smoothness parameter $\nu$, and their versions with $\nu =1/2$ (i.e. exponential kernel), $3/2$, $5/2$ and $\infty$ (i.e. squared exponential kernel) have been widely implemented [47] and built into diverse ML toolkits. The kernel function of GP can also be easily incorporated with external knowledge [49, 50]. Moreover, GP enables flexible integration with deep learning models as an independent functional layer.

The current applications of GP have been employed across various domains, including materials science, chemistry, drug design and synthetic biology (Table 1). These employments can be categorized into four main scenarios: (i) optimizing multi-composition systems, molecules, or biological sequences; (ii) optimizing process parameters for material synthesis or chemical reactions; (iii) assisting in virtual screening of large libraries; (iv) constructing simple and interpretable surrogate models for complex systems, such as cost-expensive simulations [51]. In addition, some researchers have recently employed advanced GP variants for specific practical considerations, including latent variable GP for both qualitative and quantitative inputs [52], or multi-fidelity GP [53, 54] /multi-output GP [55] for combining low-cost, low-fidelity models with a limited set of high-fidelity experiments. Finally, GP is also known to underperform in high-dimensional problems, a challenge that has spurred considerable research endeavors [21, 47].

**Table 1:** Summary of previous research on uncertainty quantification.

UQ	Scenarios	Subjects
GP	Compositions optimization	**Enzymes** [21, 56–58]
		**Fluorescent protein** [59]
		**Microbial medium** [60–62]
		Perovskites [52, 63–65]
		Battery electrolytes [66–70]
		Multicomponent alloy [31, 50, 53, 71–73]
		Polymer materials [74, 75]
		Emulsion [76]
		Atomic clusters [77]
		Nanoporous materials [78, 79]
		Multilayered materials [80, 81]
		Solar cells [52]
		Photocurable ink [82]
		Metal catalysts [83]
	Processing parameters optimization	Growth conditions of carbon nanotube [84]
		Catalytic pyrolysis conditions of plastic waste [85]
		Synthesis conditions of silver nanoparticle [86]
		Synthesis conditions of polymer fibers [87]
		Synthesis conditions of perovskite solar cell [88]
		Synthesis conditions of polypeptides [89]
		Synthesis conditions of plastic materials [90]
		Reaction conditions for flow organic chemistry [33, 34, 91–102]
		Fast-charging strategies for batteries [103, 104]
	Virtual screening	Thermoelectrics [105, 106]
		Ferroelectrics [107, 108]
		Compounds with high-melting-point [109]
		Compounds with wide band gaps [110]
		Compounds with most stable grain boundary structure [111]
		Drug molecules [36, 112–116]
		Self-assembling $\pi$-conjugated peptides [117, 118]
		Stereoselective catalysts [119]
		Organic molecule with superior charge conduction [120]
		Redox-active polymers with desired oxidation potentials [121]
	Surrogates	**Compound-kinase pairs and binding affinity** [59]
		**Sequence information and stability of G-quadruplexes** [122]
		**AAV capsids** [13]
		**Genome sequence** [123]
		**Enzymes** [21]
		Ligand binding affinity [41]
		Protein–protein interaction predictions [124]
		Experimentally controllable parameters and X-ray data point [125, 126]
		Correlation between the ratios of oil in droplet mixtures and the behavior of droplets [127]
		Material composition and phase diagram [128–130]
		All alkane molecules consisting of 4–19 carbon atoms and corresponding properties [131]
		Poisson–Schrödinger simulation process [51]
		Thin-film materials [132]
		Multicomponent alloy [31, 32, 71]
		Piezoelectrics [133]
		Ferroelectrics [134]
	Processing parameters optimization	**Genetic and metabolic pathways** [135–137]
		Synthesis conditions of inorganic quantum dot [138–141]
		Synthesis conditions of nanocrystal [142]
		Reaction conditions for flow organic chemistry [143–145]
		Synthesis conditions of perovskites [65]
	Virtual screening	Drug molecules [146–152]
		Ferroelectrics [153]
		Transition metal complexes [154]
		Compound-protein interactions [155–164]
		Compound-cell line interactions [42, 165]
	Surrogates	Interatomic potential energy surfaces [166–169]
BNN	Compositions optimization	Piezoelectrics [170]
	Processing parameters optimization	Reaction conditions for flow organic chemistry [171]
		Synthesis conditions of gold nanoparticle [172]
		Synthesis conditions of perovskite [173]
	Virtual screening	Drug molecules [35]
	Surrogates	**Identify optimal perturbations for cell-state transitions** [174]
		Identify effective drug combinations [175]
DL	Compositions optimization	**Antigen sequence** [176]
		**Enzymes** [21, 177–179]
		**Fluorescent protein** [24, 177]
		**Immunoglobulin-binding protein** [23]
		**Microbial medium** [180]
		**Antibody** [179]
		**Prime editor** [179]
		Drug molecules [181]
		Protein-specific molecules [182]
		Protein-coding DNA sequences [183]
	Processing parameters optimization	Reaction conditions for flow organic chemistry [184]
	Virtual screening	Compound-protein interactions [185]
		Transition metal complexes [186, 187]
		Drug molecules [188, 189]
	Surrogates	**Inferring single-cell expression profile from bulk-sequencing data** [190]
		**Interactions between transcription factor binding sites** [191]
		**Cell’s expression response to genetic perturbations** [27]
		**Correlation between T-cell activation and mutations on T cell receptor regions** [43]
		**Correlation between mutations in p53 genes and activities** [192]
		Molecular mutagenicity prediction [193]
		Correlation between nanopore sequencing data and labels [194]
		Protein–protein interaction predictions [195]
		Correlation between single-cell expression profiles and cell type [196]
		Correlation between cell images and cell type [197]
		Correlation between molecular representations and properties [198–200]
		Effect of solvent polarity and viscosity on the dynamical concertedness of three pericyclic reactions [201]
		Chemical compounds on subcellular localization of proteins [202]
		Correlation between experimental records and oral exposure [203]

Note. The table summarizes the algorithms, application scenarios, and research purpose. UQ, uncertainty quantification; GP, Gaussian process; BNN, Bayesian neural network; DL, deep learning-based algorithms.

In genetic engineering, GP offers accurate and straightforward modeling for combinatorial optimization scenarios involving limited data (often tens to hundreds of samples), limited parameters, and low-dimensional features. For instance, Frances Arnold’s laboratory has employed GP models to investigate the optimal combination of amino acids at four positions in the protein G domain B1 [24], or to perform recombination [56, 57] based on existing sequence fragment libraries [204, 205]. In these cases, researchers leverage the dynamically updated uncertainties of GP to allocate exploration resources, aiming to avoid local traps and rapidly reach the global optima within fewer AL cycles.

#### Bayesian neural network

The Bayesian neural network (BNN) is an extended ML model in which each parameter is represented by a distribution rather than a fixed value. It is an effective UQ model with theoretically high precisions but at higher computational costs. Noting $p\left(\boldsymbol{\theta} \right)$ as the parameters’ distribution for BNN model $f$, the training of a BNN essentially involves estimating the posterior distribution $p\left(\boldsymbol{\theta} |\boldsymbol{X},\boldsymbol{Y}\right)$ on available genotype-fitness pairs $\left(\boldsymbol{X},\boldsymbol{Y}\right)$ (Equation 6). When predicting an unexplored genotype ${\boldsymbol{x}}_{\ast}$, the distribution of $f\left({\boldsymbol{x}}_{\ast}\right)$ can be inferred analytically (Equation 7).


(6) \begin{align*} p\left(\boldsymbol{\theta} |\boldsymbol{X},\boldsymbol{Y}\right)=\frac{p\left(\boldsymbol{Y}|\boldsymbol{X},\boldsymbol{\theta} \right)p\left(\boldsymbol{\theta} \right)}{\underset{\boldsymbol{\Theta}}{\int }p\left(\boldsymbol{Y}|\boldsymbol{X},{\boldsymbol{\theta}}^{\prime}\right)p\left({\boldsymbol{\theta}}^{\prime}\right)d{\boldsymbol{\theta}}^{\prime }} \end{align*}

(7) \begin{align*} p\left(f\left({\boldsymbol{x}}_{\ast}\right)|{\boldsymbol{x}}_{\ast },\boldsymbol{X},\boldsymbol{Y}\right)=\underset{\boldsymbol{\Theta}}{\int }p\left(f\left({\boldsymbol{x}}_{\ast}\right)|{\boldsymbol{x}}_{\ast },{\boldsymbol{\theta}}^{\prime}\right)p\left({\boldsymbol{\theta}}^{\prime }|\boldsymbol{X},\boldsymbol{Y}\right)d{\boldsymbol{\theta}}^{\prime } \end{align*}

BNNs have been employed for causal inference [35, 174], and in AL packages for materials and chemistry (Table 2). Since the high computational burden of training has hindered the practical implementation, approximation algorithms are frequently employed, including Markov chain Monte Carlo, Metropolis-Hastings, Monte Carlo dropout [206], Hamiltonian Monte Carlo, Bayes by backprop [207], etc. Moreover, a promising approach for extending BNNs might be Laplace approximation, which is effective in converting traditional ML models into BNNs [208], even in domain-specific large language models (LLMs) for material discovery [209]. Based on these efforts, practical algorithms have been developed. For instance, by using Monte Carlo dropout strategy to repeatedly sample model parameters, BALD [210] and BatchBALD [211] enable the quantification of the uncertainty levels of model parameters when new samples are included.

**Table 2:** A brief summary of open-source AL packages.

Package	Surrogate Models	Application Scenarios	Webpage
Dragonfly [66, 212]	GP	SOO; MOO; multi-fidelity	https://github.com/dragonfly/dragonfly/
Phoenics [213–216]	BNN	SOO; batch-mode	https://github.com/aspuru-guzik-group/phoenics
Gryffin [171, 172, 217, 218]	BNN	SOO; batch-mode	https://github.com/aspuru-guzik-group/gryffin-known-constraints
GPyOpt [60, 61, 67, 68]	GP	SOO	https://github.com/SheffieldML/GPyOpt
COMBO [81, 106, 219]	GP	SOO	https://github.com/tsudalab/combo
CAMEO [73, 130]	GP	SOO	https://github.com/KusneNIST/CAMEO_NComm/tree/v0.1
TSEMO [33, 34, 76, 92–95, 220]	GP	MOO	https://github.com/Eric-Bradford/TS-EMO
ART [135]	Ensemble	SOO	https://github.com/JBEI/ART
ZoMBI [221]	GP	SOO	https://github.com/PV-Lab/ZoMBI
PLATIPUS [173]	BNN	SOO	https://github.com/darkreactions/platipus/
BAX [222]	GP	MOO	https://github.com/src47/multibax-sklearn
LabeMate.ML [144]	Ensemble	SOO; classification	https://github.com/tcorodrigues/ActiveLearning
sMF-BO-2CoGP [54]	GP	SOO; multi-fidelity	https://github.com/anhvt2/psi-k-tutorials-2021
GAUCHE [223]	GP	SOO	https://github.com/leojklarner/gauche
EDBO+ [224]	GP	MOO; batch-mode	https://www.edbowebapp.com/
Olympus [225]	BNN	SOO	https://github.com/aspuru-guzik-group/olympus
ChemOS [171, 214, 215, 226]	BNN, Ensemble, GP	SOO; batch-mode	https://github.com/aspuru-guzik-group/ChemOS

Note. The table summarizes the package names, models used for UQ, application scenarios, and corresponding web links. SOO, single-objective optimization; MOO, multi-objective optimization; GP, gaussian process; BNN, Bayesian neural networks; Ensemble, ensemble-based AL. All packages are designed for regression ML models by default except LabeMate.ML. Gryffin is often combined with Chimera [227], to scalarize multiple objectives into a single one. ChemOS [226] is the integration of multiple packages, including Phoenics (BNN), SMAC (Ensemble-based) [228], and Spearmint (GP) [229].

BNNs are particularly well-suited for modeling complex biological systems, offering efficient scalability to large datasets. Unlike traditional models, BNNs can flexibly capture intricate data distributions without the need for manual tuning of kernel functions or hyperparameters. For instance, Zhang *et al.* [174] applied Bayesian linear regression to model perturbations in cell-state transitions, leveraging BNNs to uncover underlying patterns in single-cell transcriptomic data and to build accurate surrogate models with minimal AL cycles.

#### Ensemble-based algorithms

Numerically estimating the uncertainty from an ensemble of multiple ML models (ensemble-based) represents one of the simplest UQ approaches. Consider pretrained ML models ${f}_k,\kern0.5em k=1,2,\dots, m$, which are distinct in parameters due to different random seeds, model architectures, or bootstrapping. When predicting an unexplored genotype ${\boldsymbol{x}}_{\ast}$, the standard deviation ${\sigma}_{\ast}$ of ${f}_k\left({\boldsymbol{x}}_{\ast}\right)$ will be numerically estimated as an approximation of uncertainty (Equation 8).


(8) \begin{align*} {\mu}_{\ast }=\frac{1}{m}\sum_{k=1}^m{f}_k\left({\boldsymbol{x}}_{\ast}\right),{\sigma}_{\ast }=\sqrt{\frac{1}{m-1}\sum_{k=1}^m{\left[{f}_k\left({\boldsymbol{x}}_{\ast}\right)-{\mu}_{\ast}\right]}^{\mathbf{2}}} \end{align*}

The simplicity and strong compatibility have made the ensemble-based approach well-suited for various practical challenges. For instance, the ensemble-based approach can be easily integrated without altering the existing state-of-the-art model structure, including those for predicting interatomic potential energy surfaces (ANI-1 potential) [166–169], drug effects (AutoQSAR) [146–148] or transcriptional regulation (Basenji2) [123]. Moreover, researchers also commonly employed ensemble-based approach for classification tasks, such as employing random forests models for predicting compound-protein interactions [155–160] (Table 1).

Despite its wide-spread applications, some researchers have raised the concerns about the potential underestimation of uncertainty. This led to the development of advanced deep ensemble algorithm [230], which has been recently applied in material science [231] and protein design [21].

Ensemble-based methods are well-suited for genetic engineering scenarios involving large-scale, high-dimensional data, and less demanding precision requirements. In such cases, uncertainty can be estimated without disrupting the research pipeline, simply by replicating original ML models at the cost of additional computational resources. For instance, Bajwa *et al.* [123] used an ensemble-based approach to estimate uncertainty in Basenji2’s predictions on genome sequences exceeding 100 k base pairs. By replicating the training processes, they found that Basenji2 exhibits overconfidence in its predictions of human reference genome sequences, but low confidence in predicting sequences with genetic variants. In conclusion, the ensemble-based approach has the potential to reveal the limitations of complex ML models and guide future biological mechanism discovery in minimal AL cycles.

#### Deep learning-based algorithms

Selecting deep learning-based (DL-based) metrics as a rough approximation of uncertainty is a simple and straightforward UQ approach. This approach typically quantifies deep learning model outputs into metrics representing uncertainty, and has been adopted in various studies [176, 181, 182] (Table 1).

One common scenario arises when the model outputs follow a categorical distribution $\hat{y}=\left[{p}_1,{p}_2,\dots, {p}_n\right],\kern0.5em \sum_{i=1}^n{p}_i=1$, where ${p}_k\ge{p}_i,i\ne k$ represents the highest probability (i.e. the predicted label is $k$). Qualitatively, the smaller the value of ${p}_k$, the greater the model’s uncertainty about the predicted label. Specifically, researchers have employed Shannon entropy and margin uncertainty for classification models [191] and log-likelihood for LLMs [232, 233].

Another common scenario involves using ML models for regression tasks. Since regression outputs cannot be directly mapped to statistical metrics like entropy, it is natural to estimate uncertainty using the expected loss of unlabeled inputs. A classic algorithm is LL4AL [234], which introduces an additional lightweight loss prediction module. This module takes multilevel features from several hidden layers of the base model as input and learns to predict the loss, trained jointly with the original task. A practical AL pipeline, muTOX-AL [193], has also recently employed an uncertainty estimation module for molecular mutagenicity prediction in drug discovery.

The expected change in model weights after adding new samples is another typical uncertainty measure for regression tasks. It represents a decision-theoretic approach by estimating future errors or weight updates. One representative algorithm is expected gradient length (EGL) [235], which measures uncertainty by the expected gradient change in the objective function after labeling new samples. In addition, BADGE [236] is a representative UQ method that integrates uncertainty estimation with diversity sampling. Specifically, it measures uncertainty by computing the gradient of new samples at the final layer and applies clustering to select the most diverse subset. Details on diversity-based sampling will be discussed in the following sections.

In genetic engineering, a common strategy is to analyze the categorical outputs of classification models. For instance, Friedman *et al.* [191] quantified uncertainty in a 4-class enhancer type predictor using entropy of class probabilities, enabling more accurate models and reducing the number of samples needed in AL cycles.

### Acquisition functions for optimal sample selection

Selecting the most informative candidate genotypes for experimentation is essential for reducing bias in training data and improving model reliability. Although both ${\boldsymbol{\mu}}_{\ast}$ and ${\boldsymbol{\sigma}}_{\ast}$ for unexplored genotype ${\boldsymbol{x}}_{\ast}$ can be quantified through various UQ approaches, their significance varies depending on specific scenario. In drug discovery, uncertain candidates are often selected to boost ML model accuracy in predicting compound-protein interactions [155, 202]. In enzyme engineering, high-prediction candidates are preferred for finding new, high-functional proteins [21, 56].

In this section, we focus on the algorithms in selecting optimal samples. The selection process differs significantly for optimizing on a single function (single-objective optimization, SOO) versus simultaneously optimizing on multiple objectives (multi-objective optimization, MOO). Another practical concern arises from specific time-consuming DBTL cycles, necessitating a selection process that improves sampling scales and diversity to minimize the number of iterations (batch-mode) [56].

To address these practical concerns, researchers in AL have proposed a series of acquisition functions for efficient sampling, tailored for various SOO, MOO and batch-mode scenarios. We will discuss these functions on their mathematical foundations, strengths, and weaknesses in the following section (Fig. 2).

#### Single-objective optimization

SOO represents the simplest optimization scenario, as model outputs can be directly compared for prioritization. A significant portion of genetic engineering research essentially involves SOO problems, such as designing proteins with a single enhanced function (e.g. binding affinity, thermostability [56] or fluorescence [177]). In the following sections, we will discuss various acquisition functions for SOO, through an example of maximizing fitness value with a GP $f$.

Although we use GP for illustration, approaches that can model ${\mu}_{\ast }$ and ${\sigma}_{\ast }$ by deep learning, such as BNN or ensemble-based methods, are also compatible with these acquisition functions. However, since these approaches often rely on numerical approximation, the accuracy of these methods may sometimes fall short of expectations. Combining the estimated ${\mu}_{\ast }$ and ${\sigma}_{\ast }$ through acquisition functions in such scenarios may introduce bias, which warrants further investigation.

#### Greedy sampling

A simple strategy is to ignore uncertainty and select genotypes solely based on model predictions, representing a purely exploitative approach aimed at rapidly acquiring viable designs. A series of genetic engineering research involves this exploitative strategy, including the design of DNA [183], proteins [177, 178], and culture mediums [180].


(9) \begin{align*} \mathrm{Exploit}\left({\boldsymbol{x}}_{\ast}\right)=\mu \left({\boldsymbol{x}}_{\ast}\right) \end{align*}

Here, we assume the sampling scale is K, and the $\mathrm{topK}$ function will represent the subset ${\boldsymbol{X}}_{\ast}^{\left(\boldsymbol{t}\right)}\subseteq{\boldsymbol{X}}_{\mathbf{cand}}$ consisting of the first K candidates with the highest acquisition function values. Throughout the subsequent sections, for all acquisition functions, ${\boldsymbol{X}}_{\ast}^{\left(\boldsymbol{t}\right)}$ will be selected via the identical $\mathrm{topK}$ screening process outlined herein, unless otherwise specified.

#### Basic balanced functions

Four commonly used acquisition functions effectively balance uncertainty and prediction: probability of improvement (PI), expected improvement (EI), upper confidence bound (UCB) [237] and Thompson sampling (TS) (Equations 10–13). These functions have demonstrated promise in material [31] and chemical engineering [91] over the past decade. Despite no significant differences in performances or applications observed previously, EI is the most favored [238].


(10) \begin{align*} {\alpha}_{\mathrm{PI}}\left({\boldsymbol{x}}_{\ast}\right)=\Phi \left(\frac{\mu \left({\boldsymbol{x}}_{\ast}\right)-f\left({\boldsymbol{x}}_{+}\right)}{\sigma \left({\boldsymbol{x}}_{\ast}\right)}\right) \end{align*}

(11) \begin{align*} {\alpha}_{\mathrm{EI}}\left({\boldsymbol{x}}_{\ast}\right)=&\left(\mu \left({\boldsymbol{x}}_{\ast}\right)-f\left({\boldsymbol{x}}_{+}\right)\right)\Phi \left(\frac{\mu \left({\boldsymbol{x}}_{\ast}\right)-f\left({\boldsymbol{x}}_{+}\right)}{\sigma \left({\boldsymbol{x}}_{\ast}\right)}\right)\nonumber\\&+\sigma \left({\boldsymbol{x}}_{\ast}\right)\phi \left(\frac{\mu \left({\boldsymbol{x}}_{\ast}\right)-f\left({\boldsymbol{x}}_{+}\right)}{\sigma \left({\boldsymbol{x}}_{\ast}\right)}\right) \end{align*}

(12) \begin{align*} {\alpha}_{\mathrm{UCB}}\left({\boldsymbol{x}}_{\ast}\right)=\mu \left({\boldsymbol{x}}_{\ast}\right)+\lambda \sigma \left({\boldsymbol{x}}_{\ast}\right) \end{align*}

(13) \begin{align*} {\alpha}_{\mathrm{TS}}\left({\boldsymbol{x}}_{\ast}\right)=\hat{f}\left({\boldsymbol{x}}_{\ast}\right) \end{align*}

Here ${\boldsymbol{x}}_{\ast }$ represents the unexplored genotype, with its uncertainty quantified. In PI and EI, $\Phi$ and $\phi$ represent the cumulative distribution function and probability distribution function of the standard normal distribution, respectively, and ${\boldsymbol{x}}_{+}=\arg \underset{x}{\max }f\left(\boldsymbol{x}\right)$ denotes the genotype with the highest predicted fitness. In UCB, $\lambda$ is a parameter that balances exploration and exploitation, empirically set high initially to prioritize exploration and gradually reduced as the optimization progresses. In TS, the $\hat{f}\left({\boldsymbol{x}}_{\ast}\right)$ is sampled from the posterior distribution of $f\left({\boldsymbol{x}}_{\ast}\right)$, for instance, $N\left({\boldsymbol{\mu}}_{\ast },{\boldsymbol{\sigma}}_{\ast}^{\mathbf{2}}\right)$ for a GP model [239]. The $\hat{f}$ will be updated iteratively as $f$ is updated. Moreover, TS is versatile, easy to compute, and also powerful in MOO.

#### Advanced look-ahead functions

Compared with basic balanced functions, look-ahead functions focus on directly evaluating the model performance improvement with additional candidates. Specifically, it quantifies the expected increase in the maximum predicted fitness of model trained with additional genotype ${\boldsymbol{x}}_{\ast}$ [240]. It is theoretically promising in scenarios where the training data $\left(\boldsymbol{X},\boldsymbol{Y}\right)$ is noisy or highly sparse, harming the reliability of model’s quality, thus decision-makers might not trust the balanced functions calculated from an unreliable model $f\mid \boldsymbol{X},\boldsymbol{Y}$, and might prefer to directly evaluate the model’s performance improvements with additional candidates. Currently, knowledge gradient (KG) and entropy search (ES) are commonly employed (Equation 14).


(14) \begin{align*} {\alpha}_{\mathrm{KG}}\left({\boldsymbol{x}}_{\ast}\right)&={E}_{p\left({\boldsymbol{y}}_{\#}|{\boldsymbol{x}}_{\ast}\right)}\left[\max \left(f\right|\boldsymbol{X},\boldsymbol{Y},{\boldsymbol{x}}_{\ast},{\boldsymbol{y}}_{\#}\right)-\max \left(f|\boldsymbol{X},\boldsymbol{Y}\right)\Big]\nonumber\\{\alpha}_{\mathrm{ES}}\left({\boldsymbol{x}}_{\ast}\right)&=H\left[f\left({\boldsymbol{x}}_{+}\right)|\boldsymbol{X},\boldsymbol{Y}\right]-{E}_{p\left({\boldsymbol{y}}_{\#}|{\boldsymbol{x}}_{\ast}\right)}\left[H\left[f\left({\boldsymbol{x}}_{+}\right)|\boldsymbol{X},\boldsymbol{Y},{\boldsymbol{x}}_{\ast },{\boldsymbol{y}}_{\#}\right]\right] \end{align*}

Here $p\left({\boldsymbol{y}}_{\#}|{\boldsymbol{x}}_{\ast}\right)=f\left({\boldsymbol{y}}_{\#}|\boldsymbol{X},\boldsymbol{Y},{\boldsymbol{x}}_{\ast}\right)$ represents the probability that unexplored genotype ${\boldsymbol{x}}_{\ast }$ has fitness value ${\boldsymbol{y}}_{\#}$ according to $f$, ${\boldsymbol{x}}_{+}$ represents the genotype with highest predicted fitness, and $H$ represents the entropy function. These look-ahead functions have been employed across various domains, including KG for shape memory alloys optimization [31, 50, 71] and ES for chemistry reaction conditions optimization [213].

#### Constrained acquisition functions

Constrained optimization represents a specific scenario where the selected candidates ${\boldsymbol{x}}_{\ast }$ can only be chosen from a subset of the total candidates ${\boldsymbol{X}}_{\mathbf{cand}}$ that satisfy the condition $\forall k\in \left\{1,2,\dots, K\right\},{c}_k\left({\boldsymbol{x}}_{\ast}\right)\ge 0$. This means that, the candidates must not only maximize $f$, but also adhere to specific constraints ${c}_1\sim{c}_K$ to address practical concerns. For instance, a food company might wish to design the best-tasting cookie while ensuring that the number of calories is below a certain level [241]. Lots of constrained acquisition functions have been designed, many of which are variants of EI, including integrated expected conditional improvement (IECI) [242] and weighted EI (wEI) [243, 244] (Equation 15).


(15) \begin{align*} {\alpha}_{\mathrm{IECI}}\left({\boldsymbol{x}}_{\ast}\right)&=\int \left[{\alpha}_{\mathrm{EI}}\left(\boldsymbol{x}\right)-{\alpha}_{\mathrm{EI}}\left(\boldsymbol{x}|{\boldsymbol{x}}_{\ast}\right)\right]h\left(\boldsymbol{x}\right)d\boldsymbol{x}\nonumber\\{\alpha}_{\mathrm{wEI}}\left({\boldsymbol{x}}_{\ast}\right)&={\alpha}_{\mathrm{EI}}\left({\boldsymbol{x}}_{\ast}\right)h\left({\boldsymbol{x}}_{\ast}\right) \end{align*}

Here ${\alpha}_{\mathrm{EI}}\left(\boldsymbol{x}|{\boldsymbol{x}}_{\ast}\right)$ indicates the EI conditioned on the assumption that ${\boldsymbol{x}}_{\ast }$ has also been included, but without making any assumptions about its true fitness value, and $h\left(\boldsymbol{x}\right)$ represents a density function that measures the probability of satisfying the constraints. The IECI function can be viewed as selecting ${\boldsymbol{x}}_{\ast }$ to minimize the EI, whereas the wEI function is more straightforward, involving the multiplication of the EI by the probability of constraint satisfaction. The constrained acquisition function is crucial for various practical scenarios, and has been applied to designing fast-charging strategies for batteries subject to the constraints of battery temperature and voltage [103, 104].

#### Multi-objective optimization

MOO represents an advanced optimization scenario that requires the simultaneous optimization of multiple functions ${f}_i,i=1,\dots, m$. The major challenges in MOO arise from the need to compare designs, as improvements of one function often come at the expense of others. For example, a chemical process that achieves low operational costs and high yield may also result in environmental destruction [92]. Directly comparing improvements in one function with sacrifices in another is problematic, because they typically have different units of measurement. To address this challenge, researchers have developed a set of well-defined mathematical symbols and theoretical frameworks, to transfer valuable insights from SOO to MOO problems [245]. We will discuss these efforts by examining an instance of maximizing multiple functions using with GPs (Equation 16).


(16) \begin{align*} \mathrm{PF}\left(\boldsymbol{X},\boldsymbol{Y}\right)&=\left\{{\boldsymbol{x}}_{\ast }|{\boldsymbol{x}}_{\ast}\in \boldsymbol{X},\nexists \boldsymbol{x}\in \boldsymbol{X},\mathrm{s}.\mathrm{t}.\kern0.5em \boldsymbol{x}\succ{\boldsymbol{x}}_{\ast}\right\} \nonumber\\ \mathrm{HVI}\left({\boldsymbol{x}}_{\ast},{\boldsymbol{y}}_{\#}\right)&=\mathcal{H}\left(\mathrm{PF}\left(\boldsymbol{X},\boldsymbol{Y}\right)\cup \left\{{\boldsymbol{x}}_{\ast},{\boldsymbol{y}}_{\#}\right\}\right)-\mathcal{H}\left(\mathrm{PF}\left(\boldsymbol{X},\boldsymbol{Y}\right)\right) \end{align*}

Here $\left(\boldsymbol{X},\boldsymbol{Y}\right)$ represents the available genotype-fitness pairs, and $\boldsymbol{x}\succ{\boldsymbol{x}}_{\ast}$ indicates that ${f}_i\left(\boldsymbol{x}\right)\ge{f}_i\left({\boldsymbol{x}}_{\ast}\right)$ for all $i=1,2,\dots, m$, with at least one inequality being strict, normally referred to as $\boldsymbol{x}$ dominate ${\boldsymbol{x}}_{\ast}$. The $\mathrm{PF}$ represents the Pareto frontiers, a set of genotypes that are not dominated by any other $\boldsymbol{x}\boldsymbol{\in}\boldsymbol{X}$. The $\mathcal{H}$ symbols hypervolume, an indicator quantifies scale of genotypes dominated by $\mathrm{PF}$, while $\mathrm{HVI}$ quantifies the improvements in $\mathcal{H}$ by selecting new data $\left({\boldsymbol{x}}_{\ast},{\boldsymbol{y}}_{\#}\right)$.

Practical acquisition functions for MOO are mainly inspired by those for SOO, including expected hypervolume improvement (EHVI) [246] derived from EI, and TS efficient multi-objective optimization (TSEMO) [220] derived from TS. While other inspirations exist (e.g. 2D-PI [154] derived from PI, EI-Centroid, EI-maximin [247] from EI), EHVI and TSEMO are most commonly employed.


(17) \begin{align*} {\alpha}_{\mathrm{EHVI}}\left({\boldsymbol{x}}_{\ast}\right)&=\underset{V_{nd}}{\int}\mathrm{HVI}\left({\boldsymbol{y}}_{\#}\right)p\left({\boldsymbol{y}}_{\#}|{\boldsymbol{x}}_{\ast },\boldsymbol{X},\boldsymbol{Y}\right)\mathrm{d}{\boldsymbol{y}}_{\#} \nonumber\\{\alpha}_{\mathrm{TSEMO}}\left({\boldsymbol{x}}_{\ast}\right)&=\mathrm{HVI}\left({\boldsymbol{x}}_{\ast},\left\{{\hat{f}}_1\left({\boldsymbol{x}}_{\ast}\right),{\hat{f}}_2\left({\boldsymbol{x}}_{\ast}\right),\dots, {\hat{f}}_m\left({\boldsymbol{x}}_{\ast}\right)\right\}\right) \end{align*}

Here ${V}_{nd}$ denotes all potential fitness values that are not dominated by $\mathrm{PF}\left(\boldsymbol{X},\boldsymbol{Y}\right)$, while $p\left({\boldsymbol{y}}_{\#}|{\boldsymbol{x}}_{\ast },\boldsymbol{X},\boldsymbol{Y}\right)$ is the probability of unexplored genotype ${\boldsymbol{x}}_{\ast }$ having fitness values ${\boldsymbol{y}}_{\#}$ according to ${f}_i,i=1,\dots, m$. In TSEMO, ${\hat{f}}_i\left({\boldsymbol{x}}_{\ast}\right)$ is sampled from posterior distribution from ${f}_i\left({\boldsymbol{x}}_{\ast}\right)$, and all ${\hat{f}}_i$ will be updated iteratively and simultaneously. Both EHVI [72, 87, 90, 143, 186, 187, 224] and TSEMO [33, 34, 76, 92–95, 220] have been validated across diverse materials and chemical studies, suggesting potential adoptions in genetic engineering.

#### Batch-mode optimization

A key challenge in genetic engineering is the lengthy process of building and testing, which can last for days or even months. This process includes DNA synthesis, polymerase chain reaction (PCR) amplification, assembly, sequencing, and transformation, etc. To reduce DBTL cycles, researchers have proposed batch-mode acquisition functions that enable the parallel selection of multiple batches, allowing for the exploration of diverse uncertain regulatory patterns or the exploitation of multiple potential fitness peaks simultaneously.

Various acquisition functions have been adapted for batch-mode settings. Among the fundamental acquisition functions, PI, EI, UCB, and TS have all been extended to handle batch recommendations. However, each has its own characteristics in parallel settings. The acquisition function of PI, while computationally efficient and simple to implement through batch-mode selection, tends to be overly exploitative and often struggles with maintaining batch diversity, potentially getting stuck in local optima. The EI acquisition function offers a better balance between exploration and exploitation with strong theoretical foundations, particularly through variants like q-EI [248], but becomes computationally expensive for large batch sizes and may require Monte Carlo sampling for exact computation. The acquisition function of UCB provides strong theoretical guarantees and naturally extends to parallel settings through GP-UCB-PE [249], offering explicit exploration-exploitation trade-off through its hyperparameter, though it requires careful tuning and may be overly exploratory. TS emerges as perhaps the most natural choice for parallelization, maintaining diversity through random sampling and offering computational efficiency for multiple recommendations without requiring additional hyperparameters, although it can suffer from higher variance and potentially slower convergence [250]. In terms of practical implementation, TS scales best with batch size with $\mathrm{O}(n)$ complexity [250], followed by UCB with $\mathrm{O}(nlogn)$ [249], while PI and especially EI become more challenging with larger batches. For smaller batch sizes, EI-based methods are recommended if computational cost is acceptable, while for medium to large batch sizes, TS offers the best practical choice due to its natural parallelization and scalability.

Beyond the classical acquisition functions, researchers have developed more sophisticated approaches specifically designed for batch-mode optimization. Notable among these are *q*-EHVI [90, 224, 251], which extends the concept of EI to handle multiple objectives simultaneously in the parallel setting. Another innovative approach is cluster-based Sørensen–Jaccard sampling [252], which leverages clustering techniques to maintain diversity in batch selections. The Barriers to their adoptions in genetic engineering are decreasing, thanks to the growing affordability of high-throughput experimentation.

### Acquisition functions for model accuracy improvement

Efficient sample selection to improve model accuracy is another key task. In contrast to strategies focused on identifying the most informative samples, this task emphasizes the representativeness of newly acquired data. Samples are typically selected in batches to maximize predictive performance with minimal AL iterations and samples. Some methods utilize uncertainty estimates (Section 3.2), while others prioritize sampling diversity, enabling a more flexible range of algorithms (Fig. 2).

#### Sampling with highest uncertainty

Iteratively selecting the most uncertain genotypes represents an exploratory strategy aiming at achieving a more comprehensive coverage of biological insights. For instance, prior to the deep learning era, Tong *et al.* [253] and Schohn *et al.* [254] selected samples near the SVM decision boundary for AL. This approach builds a highly representative training set, enabling accurate ML models with minimal labeling, and has been widely applied in biological tasks such as modeling compound–protein interactions for drug discovery [155–160]. The acquisition function for this process can be formulated as:


(18) \begin{align*} \mathrm{Explore}\left({\boldsymbol{x}}_{\ast}\right)=\sigma \left({\boldsymbol{x}}_{\ast}\right) \end{align*}

As aforementioned, selecting the most uncertain samples, using metrics such as Shannon entropy, has proven effective in improving classification models for practical genetic engineering tasks, such as modeling interaction patterns of transcription factor binding sites [191] and mutational effects in T-cell receptor regions [43]. Furthermore, for nonclassification models, strategies like LL4AL, which select samples with the highest expected loss, have also demonstrated strong performance, outperforming various methods including entropy-based sampling.

Moreover, the representational ability of newly acquired samples for unknown spaces is crucial. The aforementioned strategy focuses on labeling the least confident samples. However, in a batch setting, this can lead to a scenario where the data within the batch are similar or nearly identical, resulting in inefficiency.

#### Diversified sampling

To improve sampling efficiency, we can select samples to enhance batch diversity. Diversifying the sampling of the unknown data’s distribution complements uncertainty-based methods. This means focusing on the representativeness of the samples.

An effective approach is to employ clustering strategies, such as clustering the model’s feature maps to diversify sampling within the cluster space, thereby preventing over-investment in particularly dominant classes. The k-determinantal point process (k-DPP) algorithm [255] samples k points from a set with probabilities proportional to the determinant of their Gram matrix, effectively selecting a diverse subset, and has been applied to AL tasks like image classification [256]. The k-MEANS++ algorithm [257] samples centroids iteratively, assigning each sample a probability proportional to the squared distance from its nearest selected centroid, thereby promoting the selection of highly representative samples. This inspired BADGE [236], which first quantifies uncertainty using gradients, then clusters them via k-MEANS++ to select centroids, achieving a balance between uncertainty and representativeness.

The Core-set algorithm [258, 259] is another important strategy. It selects a set of samples (core set) that maximally covers the distribution of the unlabeled data. The core idea is to solve the k-center problem, which aims to find k sample points that minimize the maximum distance from any unlabeled point to its nearest center. Specifically, the distance metric can be derived from the model, such as the L2 distance between feature maps from fully connected layers.

## Implementations of active learning in genetic engineering

The expanding arrays of AL approaches has led to significant practical implementations across various domains, giving rise to self-driving laboratories (SDLs) and manufacturing processes that facilitate autonomous knowledge integration, experimentation, and hypothesis generation [28, 260, 261]. Despite these inspiring successes, the scattered implementations of AL in genetic engineering have not yet been discussed, organized, or extended, giving the impression that the field is still at a nascent stage. This vicious cycle strongly arises from researchers’ concerns about whether AL approaches that are efficient in other scenarios will also perform with highly-specific, complex biological data. The key to breaking this cycle lies in providing confidence through well-organized implementations across a range of biomolecules and experimental settings, enabling researchers to recognize the significant opportunities they might be overlooking, and to contribute their valuable efforts to the research community.

**Figure 3:**
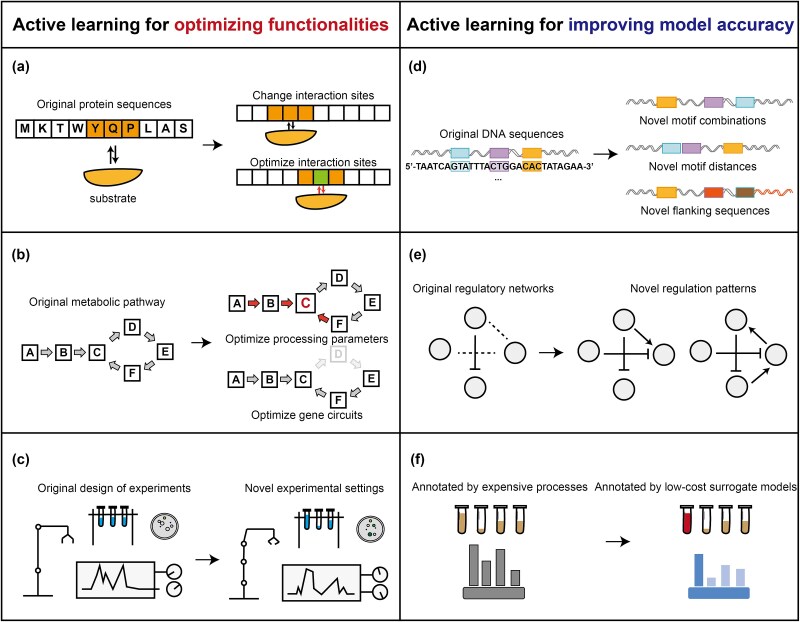
Current implementations of AL in synthetic biology. Left, AL for optimizing the functionalities, including (a) protein engineering (b) metabolic pathways optimization (c) increasing the efficiencies of experiments. Right, AL for improving the accuracy of ML models, including (d) interpreting DNA regulatory patterns (e) modeling GRNs (f) building surrogate models for annotation.

In the following sections, we present organized efforts regarding AL in genetic engineering across diverse scenarios, covering protein engineering [13, 21, 24, 43, 56–59, 176–178], optimization of metabolic pathways [135, 136, 262], improvements in experimental efficiency [60–62, 180], interpretation of DNA regulatory patterns [122, 123, 191, 192], modelling of GRN [27, 174], and reduction of annotation efforts [190] (Fig. 3).

### Active learning for optimizing designs with enhanced functionalities

Advancing functionalities (e.g. yield, stability, and selectivity) beyond the limits of natural evolution is a paramount mission for researchers, with implications that extend beyond academia and provide significant benefits to industries such as biomanufacturing and precision medicine. Currently, the expanding array of AL implementations has covered the optimizations across diverse functionalities in biomolecules, gene circuits, or experimental protocols. In this section, we will organize these implementations and highlight some influential or near-term cases to discuss their engineering scenarios, AL approaches, and functionality improvements.

#### Protein engineering

The implementations of AL can be traced back to the pioneering efforts of Frances Arnold’s lab, which stemmed from reducing labors in directed evolution of cytochrome P450s [56]. For the vast protein space with extremely scarce functional proteins ($1/{10}^{11}\hbox{--} 1/{10}^{77}$), extracting solutions from rational design or chimeragenesis are not tractable. Instead, GP models coupled with batch-mode UCB functions were employed. The final EXPc5 has a thermostability increase of 5.3 C than previously identified thermostable P450s. Following this early-stage attempt, AL has been expanded to diverse types of proteins or functional domains, including green fluorescent protein [59, 177], TEM-1 β-lactamase [177, 178], fatty acyl reductases [58], protein G domain B1 [21, 24], β-subunit of tryptophan synthase [21], AAV capsids [13], heavy-chain complementarity-determining region 3 [176], and T cell receptor [43]. These implementations not only broaden the application scenarios, but also align with a growing trend of integrating advanced deep learning techniques, including zero-shot learning [24], encoding by LLMs [21, 24, 59, 177–179], deep ensembles [21], and deep generative networks [176]. Moreover, the collaboration between AL and automation techniques has recently culminated [57], leading to the development of an SDL that covers all labor-intensive processes throughout the entire DBTL cycle. Recently Jiang *et al.* proposed EVOLVEpro [179], which successfully extended the AL method to the efficient design of antibodies, CRISPR nucleases, prime editors, and RNA polymerases.

#### Metabolic pathway optimization

Optimizing the metabolic pathways mainly involves engineering gene circuits or process parameters (e.g. temperature, composition, and induction times) to overexpress productive pathways and suppressing competing ones [263]. One line of research exploits AL to tackle the lengthy numerical simulations involved in designing large-scale genetic pathways [262], resulting in successful optimizations for complex pathway with 27 candidate architectures. Another line of research focused on automated optimization of process parameter with limited training instances. They proposed an ensembled-based AL package ART [135, 137], leading to a significant increase in tryptophan productivity (106%) in the aromatic amino acid pathway of *Saccharomyces cerevisiae*. Moreover, the MEITS framework, developed by Pandi *et al.* [136], exemplifies deep collaboration between genetic engineering, AI and automation. With ensemble-based algorithms and UCB strategies, MEITS enables the optimization of combinations of transcription and translation units (12-fold increase), as well as pathways such as LacI gene circuits (34-fold) and the crotonyl-CoA/ethylmalonyl-CoA/hydroxybutyryl-CoA (CETCH) cycle (6-fold in efficiency, 10-fold in productivity).

#### Improving experimental efficiency

The efficiency of experiments is an enduring pursuit for scientists, as some tips in improving efficiency have revolutionized the entire research community and even industry (e.g. phase diagrams, Karnaugh maps and double-entry bookkeeping). In genetic engineering, AL has recently demonstrated its potential to enhance experimental efficiency through optimizing mediums compositions. Yoshida *et al.* [61] and Watanabe *et al.* [60] have employed GPyOpt package along with the acquisition functions of EI and UCB to optimize medium compositions for Escherichia coli (*E. coli*), resulting in increased green fluorescent protein expression level of 2.13-fold and 1.4-fold, respectively. Similar studies have also been conducted to optimize culture media for mammalian cells [180] and liquid media [62]. Despite employing different AL approaches (gradient-boosting decision tree and GP), both studies achieved significant improvements over baseline media after no more than three DBTL cycles.

### Active learning to reduce bias between models and experiments

Bias in model estimation and experimental outcomes stems from noisy or imbalanced data, which is common in areas like single-cell RNA sequencing [264], medical imaging [265], and protein–protein interactions [266]. Such bias presents a significant barrier to interpreting intrinsic biological mechanisms, limiting the ability of researchers to tackle cutting-edge challenges in fields like immunology and aging. AL has begun to address this issue, with implementations spanning various tasks, including the interpretation of biological sequences, modeling GRNs, inferring responses to perturbations, and building surrogate models for annotation. In this section, we will provide a brief overview of these notable progresses, highlighting their innovations in both technology and application.

#### Interpreting regulatory patterns

Decoding the complex cis- and trans-regulatory patterns underlying gene expression has long been a challenging issue, due to the immense diversity of potential motif combinations, inter-motif spacing, and sequence contexts. AL has offered promising strategies to reduce the data required for training ML models in this domain. Early efforts in 2009 used AL to interpret sequence features affecting G-quadruplex stability [122] and identify cancer-rescue mutants in p53 gene [192]. Another wave of AL implementations has been sparked by improvements in deep learning techniques over the past decade. Friedman *et al.*’s study in 2023 focused on interpreting cis-regulatory “grammars” in photoreceptors [191], investigating how the spatial configurations of transcription factor binding sites influence sequence activities, distinguishing them as strong enhancers, weak enhancers, inactive sequences, or silencers. Using a support vector machine classifier alongside entropy—and margin-based AL functions, they achieved success only after three rounds of selection, evaluating a total of 35 832 sequences. Moreover, recent efforts have extended AL for other challenging tasks, such as identifying transcription factors involved in cell differentiation from single-cell RNA sequencing datasets [267], and quantifying uncertainty in predictive models for kb-level genomic sequences [123].

#### Modeling gene regulatory networks

The modeling of gene regulatory networks (GRNs) has been a topic issue in system biology, largely due to the sparsity of connections and the complexity of nonlinear regulatory mechanisms [268], necessitating integrations of advanced ML techniques tailored for GRNs in AL implementations. In 2023, Zhang *et al.* [174] have developed an AL framework for causal inference specified for GRNs, where the GRNs is modeled by a directed acyclic graph, with nodes denoting genes and edges encoding interventions. Their framework incorporates BNN model and a novel causal integrated variance acquisition function, and optimally intervenes on 36 genes in human melanoma cells with no more than 600 instances. Another AL framework ITERPERT [27] integrated with the kernelized multi-modal priors has achieved 3-fold speedup over the best baseline, leading to lower costs in modeling cellular responses to new genetic perturbations.

#### Building surrogate models for annotation

In practical implementations, data annotation processes like simulations, measurements, and calculations are sometimes labor-intensive and costly. Surrogate models can replace these processes, mimicking their input–output relationships with a simpler model, thus significantly reduce computational costs and provide approximative predictive capabilities. AL has been employed for accelerating the development of surrogate models (e.g. for Poisson–Schrödinger simulation [51], interatomic potential energy surfaces [166–169] and nanopore sequencing [194]). For genetic engineering, scSemiProfier [190] is the recent implementations for inferring the single-cell profiles from bulk-sequencing data. With a VAE-GAN based model and iteratively selecting the most informative candidates, scSemiProfier developed a precise surrogate for human iPSC-derived iMGL cells, reaching a Pearson correlation of 0.997 and cutting costs from $37 500 to about $21 750.

Practical strategies for improving efficiency.

Given the trend of increasing AL implementations, whether their efficiency can be improved with better approaches or configurations is warranted, necessitating the specified benchmarking to inform such improvements.

One line of research focuses on how different protein encodings influence the optimization efficiency. Wittmann *et al.* [24] selected encodings derived from eight language models (e.g. TAPE transformer [269], UniRep [270], ESM1b [271]) and performed 2000 rounds of simulations for each encoding considered. Encodings from Georgiev [272] and multiple sequence alignment (MSA) Transformer [273] outperform one-hot encodings in max fitness achieved, highlighting the significance of choosing encodings with physicochemical information (e.g. hydrophobicity, volume, mutability) or evolutionary information from multiple sequence alignments. Another innovative attempt comes from Frances Arnold’s lab, where they have benchmarked encodings against the latest progress in AL and deep learning [21]. In addition to one-hot and Georgiev encodings, they also introduced encodings from AAIndex database [274] and language model ESM2 [275], focusing on the collaboration of encodings and diverse UQ models, including ensembles of boosting models, ensembles of deep neural networks, GP and deep kernel learning [276]. The results suggest that high-dimensional encodings (20 for one-hot, 1280 for ESM2) are best paired with deep learning UQ models (i.e. ensembles of deep neural networks, and deep kernel learning), while nondeep learning UQ models (i.e. ensembles of boosting models, and GP) work well with low-dimensional physicochemical encodings (4 for AAIndex, 19 for Georgiev).

Attempts have also been made in designing informative training datasets [24]. The barriers to efficient AL lie in the fact that most genotypes are typically associated with low fitness, thus constructing an initial training dataset by reducing low-fitness data (50% of total >0.011) has led to a 19% improvement in max fitness achieved. However, this strategy fails when prior knowledge of fitness is lacking. To address this challenge, researchers have recognized the correlation between evolutionary and biophysical rules in protein sequences and fitness, and have employed three zero-shot strategies to filter mutations with potential fitness improvements, including EVmutation [277], mask filling using the MSA Transformer [273], and Triad ΔΔG calculations.

One major practical concern is the limited implementations of AL to biomolecules like RNA and glycans, representing a missed opportunity to address challenges in these focus areas. For instance, the optimization of 5’ untranslated region (UTR) sequences to improve protein expressions has been a long-standing interest [278–280]. Therefore, we encourage further implementations to fully leverage the potential of AL. Moreover, with the trend of increasing collaborations between synthetic biologists and AL researchers, we encourage further cross-domain communications to avoid applying outdated algorithms or mismatched models in genetic engineering, or neglecting specific practical concerns when developing advanced AL approaches.

## Outlooks

The integration of AL can significantly revolutionize traditional genetic engineering practices. It assists researchers throughout the DBTL cycles, enables reducing labors in building accurate ML models, and identifying the most valuable candidates for highest potential functionalities. By carefully selecting efficient UQ and sampling strategies, AL-assisted DBTL cycles have been proven effective in designing proteins and metabolic pathways with improved functionalities, enhancing experimental efficiency, as well as in learning gene regulatory patterns, modeling GRNs, and building surrogate models for annotation.

In the following, we will further highlight pivotal integrations of AL with expertise from related domains to advance genetic engineering, including cross-domain research, foundation models and automation techniques. We will demonstrate the technical enhancements enabled by these integrations and discuss how they address the practical considerations or unlock potential frontiers in genetic engineering.

### Integration of cross-domain research

The limited implementations of AL in specific genetic engineering scenarios (e.g. MOO, multifidelity, batch-mode) are restricting broader applications. To address this challenge, researchers can draw on a wealth of well-established practices in adjacent domains, including materials science [260, 261, 281–283], organic chemistry [261, 284], drug discovery [285–288], robotics, dynamics, and computer vision.

For instance, a major challenge in genetic engineering is that, although AL can reduce annotation demands, the process often remains time-consuming and costly, especially in tasks requiring expert knowledge. The integration of biological data from a variety of sources with diverse fidelity levels holds the potential to significantly reduce experimental costs. It might be hard for a researcher to find referable case studies related to genetic engineering, but wealthy cases exist in developing alloys [53, 72], nanoaggregates [117] and fast-charging protocols for batteries [37]. Another potential comes from employing batch-mode acquisition functions to select information-rich candidates, to reduce the number of iterations needed. The batch-mode AL implementations in genetic engineering have not advanced significantly for over a decade, whereas those in material science and chemistry have been continuously evolving [72, 90, 91, 169, 224, 252, 289], covering advanced functions like q-EHVI. We have organized the packages provided from these cross-domain studies, to facilitate the migration of these valuable practices (Table 2).

These cross-domain studies not only provide valuable guidance but also inspire researchers to form analogies between biological scenarios and other adjacent fields. For instance, if an AL approach is effective in optimizing chemical reactions, could it also be migrated to optimizing metabolic pathways? Could researchers validate their novel specific AL approach on well-established benchmarks from material sciences? It’s inspiring to imagine cross-domain front-line researchers collaborating together to identify commonalities and address their unique challenges. Through extensive communication and brainstorming, breakthroughs might be unlocked, which were once driven by creative ideas and lengthy investigations.

### Integration of foundation models

Recent AL implementations have not fully matched the progress in ML techniques. Foundation models are domain-specific LLMs that learn intrinsic grammars and knowledge through pretraining on large-scale and diverse datasets, thereby demonstrating strong performance and adaptability in various downstream tasks. There are series of biological LLMs developed for diverse biological data, including for single-cell transcriptomics profile [290, 291], proteins [292], DNA [4, 293] and RNA sequences [294].

The integration of foundation models can significantly advance AL for genetic engineering. For instance, ESM model trained on a large protein database [295], can score the likelihood of novel mutations, thus filtering out a large amount of ineffective labor for protein engineering [296]. Therefore, foundation models are capable of conducting digital experiments, leveraging comprehensive domain knowledge rather than only relying on uncertainties estimated from local datasets, and thus can further analyze the effectiveness of sequences designed by AL. Moreover, highly dissimilar biological sequences can be functionally similar, while the well-trained foundation models are capable of embedding such functionally similar sequences into close proximity. Therefore, we believe that introducing foundation models will significantly augment biological data, enhance the establishment of intrinsic data associations, improve the quantification of uncertainties, and ultimately further accelerate the AL-assisted DBTL cycles.

### Integration of automation techniques

The deeper integrations of automation techniques will pave the way for intelligent SDLs in genetic engineering. Although past efforts have yielded successful implementations of AL-assisted SDLs in material science [297], chemistry [261], and recently in genetic engineering [57, 136], these SDLs largely treat AL as merely an optional decision-making algorithm, which has not liberated researchers from burdensome, repetitive and monotonous tasks, and remains a distance from true intelligence. An idealized intelligent SDLs should enable researchers to simply “set it and forget it” [298]. This implies that, with basic targets and parameters settings, entire pipeline of experimental processes could run automatically, ranging from complex ones such as sequence synthesis, cells culturing, and measurements, to more routine processes like reagent procurement, equipment cleaning, autoclaving and even waste management. AL has a long history of effectiveness in developing cost-effective, high-precision equipment, with recent notable successes in computer vision (object detection, image segmentation) [299] and robotics (e.g. control, localization, mappings) [300], thus enabling intelligent SDLs affordable in reality.

A key challenge in automated experiments is determining when to stop AL cycles. Without clear stopping criteria, the process risks over-labeling, leading to wasted resources, or stopping prematurely, which may compromise model performance. Vlachos *et al.* [301] suggested stopping when the remaining unlabeled data consistently contradict the information already acquired by the model. Yu *et al.* [302] proposed halting when the gradient of the loss function, used to assess the influence of new samples, falls below a predefined threshold. Although theoretical advancements continue to emerge [303, 304], these algorithms are mainly tested in limited models and datasets, thus the question of when to stop remains an open challenge, requiring further empirical validation in real-world applications.

In the more distant future, AL holds the potential to automatically organize complex information and generate biological knowledge [28, 298], paving the way for accelerating innovation. An ideal workflow would involve collecting extensive prior knowledge, raw data, research articles, and laboratory protocols, as well as translating the learned parameters of machine learning models into human-readable mathematical equations, natural language, or graphics using interpretable algorithms. To reduce dependence on human intervention, AL could play a key role in developing proficient AI assistants capable of automatically collecting the most valuable knowledge and interpreting ML models, thereby facilitating the autonomous discovery of intrinsic biological mechanisms. We believe that as research communities and industries increasingly adopt and refine AL approaches, the future where researchers are liberated from unnecessary burdens and can fully focus on driving innovations is just a matter of time.

**Key Points**

- Active learning can help in optimizing functionalities of biomolecules, as well as bridging the gaps between model predictions and experimental outcomes.
- Active learning consists of two major steps: uncertainty quantification and acquisition of candidates.
- The further integration of active learning with cross-domain research, foundation models, and automation techniques can further address practical limitations and advance genetic engineering frontiers.

## Data Availability

None declared.
